# Real-World Clinical Outcomes and Biopsy Patterns of Older Patients with Unresected Non-Small-Cell Lung Cancer Treated with Primary Stereotactic Body Radiotherapy

**DOI:** 10.3390/jcm14238604

**Published:** 2025-12-04

**Authors:** Pragya Rai, Su Zhang, Yan Song, Chi Gao, Anya Jiang, Jiayang Li, Peixi Jiang, James Signorovitch, Ashwini Arunachalam, Andrew Song, Ayman Samkari, Megan E. Daly

**Affiliations:** 1 Merck & Co., Inc., 126 East Lincoln Ave, Rahway, NJ 07065, USA; ashwini.arunachalam@merck.com (A.A.); andrew.song@merck.com (A.S.); ayman.samkari@merck.com (A.S.); 2 Analysis Group, Inc., 111 Huntington Ave 14th Floor, Boston, MA 02199, USA; su.zhang@analysisgroup.com (S.Z.); yan.song@analysisgroup.com (Y.S.); chi.gao@analysisgroup.com (C.G.); xinyi.jiang@analysisgroup.com (A.J.); jiayang.li@analysisgroup.com (J.L.); peixi.jiang@analysisgroup.com (P.J.); james.signorovitch@analysisgroup.com (J.S.); 3 University of California-Davis Comprehensive Cancer Center, 2279 45th St., Sacramento, CA 95817, USA; medaly@ucdavis.edu

**Keywords:** clinical outcomes, unresected NSCLC, lung biopsy, non-small-cell lung cancer, real world evidence, stereotactic body radiotherapy, survival

## Abstract

**Background/Objectives**: We describe the real-world survival and utilization of lung biopsy in Medicare patients with unresected stage I-IIB (N0) non-small-cell lung cancer (NSCLC) receiving primary stereotactic body radiotherapy (SBRT) in the US. **Methods**: Patients (aged ≥66 years) with unresected stage I-IIB (N0) NSCLC who received primary SBRT were identified in the SEER-Medicare database (2007–2020) and followed from SBRT initiation until death/data end. Outcomes included overall and disease-stage-specific real-world event-free survival (rwEFS), overall survival (OS), lung cancer-specific cumulative incidence of death, and time to death or distant metastasis (TDDM). rwEFS, OS, and TDDM were described using Kaplan–Meier analysis. Median times from lung biopsy to SBRT were summarized. **Results**: Of 3014 patients (median follow-up: 2.9 years), 2302 (76.4%), 454 (15.1%), 168 (5.6%), and 90 (3.0%) had stage IA, IB, IIA, and IIB disease, respectively. The mean age at diagnosis was 77.3 years, 37.7% were male, and 86.9% were White. Overall, the 5-year rwEFS rate was 23.8% (median 26.2 months), the 5-year OS rate was 42.3% (median 48.9 months), and the 5-year lung cancer-specific cumulative incidence of death was 25.3%. rwEFS and OS rates declined with more advanced disease stage at diagnosis. Most patients (90.1%) underwent lung biopsy within 12 months before SBRT. **Conclusions**: Among older US patients with unresected NSCLC receiving SBRT, prognosis remains limited, with many deaths due to non-lung cancer causes. Recurrence and survival were lower among subgroups with more advanced disease. These findings benchmark real-world outcomes for future studies assessing novel strategies in this patient population.

## 1. Introduction

Despite steady declines in cancer mortality over the past 30 years, lung cancer remains the leading cause of cancer deaths worldwide [1]. In the United States (US), it has been estimated that 235,000 new lung cancer cases were diagnosed and more than 125,000 lung cancer-related deaths occurred in 2024 [2], underscoring the heavy clinical, humanistic, and economic burdens of this malignancy. Over 85% of all lung cancer cases are with non-small-cell lung cancer (NSCLC) [3], among which the most common histological subtype is adenocarcinoma (40% of the cases), followed by squamous cell carcinoma (25–30%) and large cell carcinoma (5–10%) [4].

Stereotactic body radiotherapy (SBRT) is a noninvasive treatment that delivers precise, high dose-per-fraction radiation to extracranial tumors using sharp dose gradients and is the recommended course of treatment for patients with inoperable stage I-II (N0) NSCLC and those with NSCLC who have elected not to undergo resection [5,6]. SBRT is well-tolerated and provides excellent local tumor control, exceeding 90% after 3 years [5,7,8], although rates of regional and distant recurrence are over 30% after 5 years of follow-up [9,10]. Similar results were reported in studies focusing on older patients with early-stage NSCLC [11,12,13]. However, existing studies are based on outdated data, data from single-center studies, and/or focused on patients with stage I disease at initial diagnosis, while contemporary data on clinical outcomes associated with SBRT in stage I-II NSCLC is scarce, with one recent large-scale retrospective study focusing on the impact of disease recurrence in early-stage NSCLC reporting that recurrence after SRBT was associated with worse survival compared with no recurrence [14]. Additionally, patients with unresected NSCLC who are candidates for primary SBRT are recommended to first undergo biopsy for diagnosis confirmation when clinically feasible [15]. However, the multidisciplinary team managing the patient may sometimes conclude that the risk of invasive biopsy to confirm the diagnosis outweighs the risk of empiric treatment with SBRT. As such, understanding the real-world utilization of lung biopsy is important for benchmarking these treatment patterns in recent clinical practice. However, there is limited real-world data on the use of lung biopsy among patients with unresected stage I-II (N0) NSCLC receiving primary SBRT.

To address these gaps in the literature, this study aimed to comprehensively describe the real-world survival outcomes, as well as utilization of lung biopsy, in older patients with stage I-II (N0) NSCLC treated with primary SBRT in the US, overall and by stage at initial diagnosis. The clinical outcomes following SBRT included overall survival (OS), real-world EFS (rwEFS), cumulative incidence of lung cancer-specific death, and time to distant metastasis or death (TDDM), overall and by disease stage at initial NSCLC diagnosis.

## 2. Materials and Methods

### 2.1. Data Source and Study Design

A retrospective observational analysis was conducted among patients with newly diagnosed unresected stage I-IIB (N0) NSCLC who received primary SBRT after initial NSCLC diagnosis. Data were drawn from the Surveillance, Epidemiology, and End Results (SEER)-Medicare database (2007–2020), which included all Medicare-eligible persons in the SEER registry diagnosed with cancer through 2019 and their Medicare claims through 2020.

The index date was defined as the SBRT initiation date, and the 12 months prior to index was defined as the baseline period. Patients were followed from the initiation of SBRT until the earliest of death; end of Medicare Part A, B, or D eligibility; or end of data availability on 31 December 2020.

All data were compliant with the Health Insurance Portability and Accountability Act. This study was exempt from Institutional Review Board oversight because it involves only the secondary use of de-identified patient records, as dictated by Title 45 Code of Federal Regulations (45 CFR 46, 101(b)(4)). The study was conducted in accordance with the principles of the Declaration of Helsinki.

### 2.2. Patient Selection

**Figure 1:**
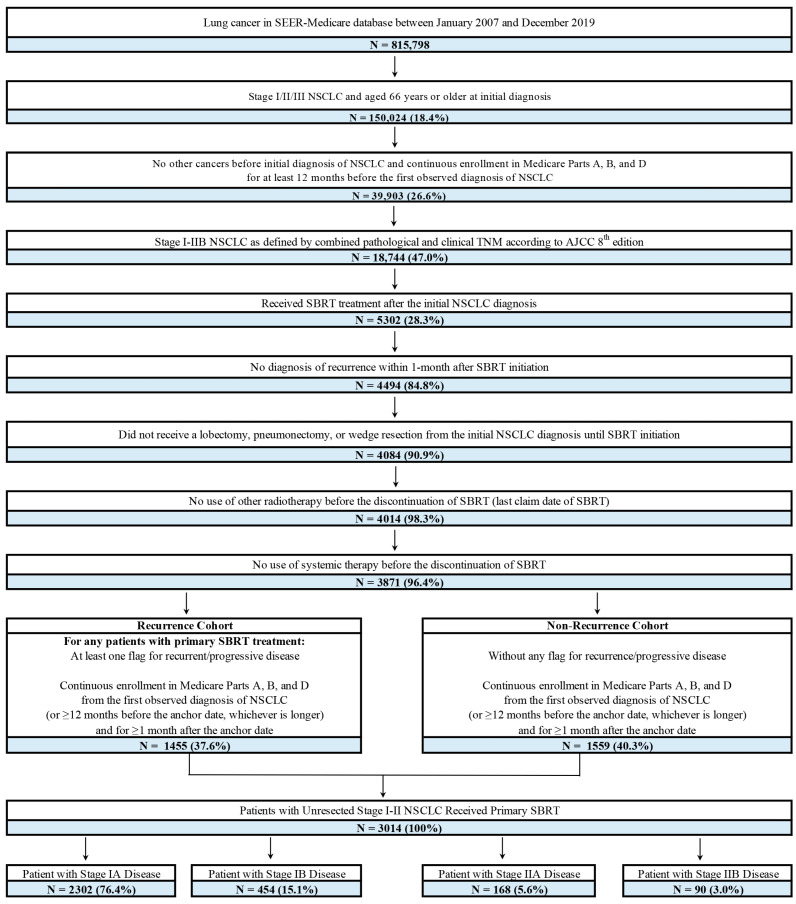
Sample selection and patient cohorts. Abbreviations: AJCC, American Joint Commission on Cancer; NSCLC, non-small-cell lung cancer; SEER, Surveillance, Epidemiology, and End Results; SBRT, stereotactic body radiotherapy; TMN, tumor, node, metastasis staging system. Note: for patients with recurrence, the anchor date was the date 30 days before the first recurrence indicator. For those without recurrence, the anchor date was assigned randomly according to the distribution of time between the date of primary SBRT initiation and the anchor date for patients from the recurrence cohort, such that the continuous enrollment duration post-primary SBRT initiation duration was comparable between the two cohorts.

Figure 1 presents the patient selection flowchart. Patients were included in the study if they (1) had a recorded NSCLC diagnosis in the SEER registry between 2007 and 2019; (2) were aged 66 years or older at initial NSCLC diagnosis; (3) had no other cancers before initial NSCLC diagnosis and had continuous Medicare enrollment for ≥12 months before the first NSCLC diagnosis; (4) had stage I-IIB (N0) disease based on the American Joint Committee on Cancer (AJCC) 8th edition tumor node metastasis (TNM) staging criteria [16]; and (5) received primary SBRT within 6 months after initial diagnosis of NSCLC. The diagnosis and procedural codes are presented in Supplementary Table S1.

Patients were excluded if they had received lobectomy, pneumonectomy, or a wedge resection/segmentectomy from the initial diagnosis of NSCLC until SBRT initiation; had diagnoses of recurrence within 30 days of initiating SBRT; or used other radiotherapy or systemic therapy before the discontinuation of SBRT.

Patients were stratified into 4 cohorts by stage of initial NSCLC diagnosis.

### 2.3. Outcomes and Measures

The study outcomes and measures included rwEFS, OS, cumulative incidence of lung cancer-specific death, TDDM, and lung biopsy patterns. rwEFS was defined as the time from the index date to first recurrence or death, whichever occurred first, wherein recurrence was defined as a diagnosis of secondary malignancy occurring ≥30 days after initiation of primary SBRT, and/or the receipt of subsequent NSCLC treatments for NSCLC (including surgery, radiotherapy, and chemotherapy) ≥30 days after the end of SBRT. OS was defined as the time from the index date to death. TDDM was defined as the time from the index date to distant metastasis or death (from any cause), whichever occurred first. Patients were censored at the earliest of loss of follow-up or end of data availability. When estimating cumulative incidence of lung cancer-specific death, competing risk of death due to other causes was adjusted using the ‘cuminc’ function in R [17].

### 2.4. Statistical Analyses

Baseline demographic (age, sex, race/ethnicity) and clinical characteristics (histology, index date, and Charlson Comorbidity Index [CCI] score) were described for patients overall and by disease stage. Continuous variables were summarized using means and standard deviations (SD); categorical variables were summarized using frequency counts and percentages. rwEFS, OS, and TDDM were described using Kaplan–Meier curves, overall and by disease stage. Cumulative incidence of lung cancer-specific death was summarized for the overall patient population and by disease stage. For lung biopsy treatment patterns, the proportions of patients receiving each type of lung biopsy were summarized. The mean and median times from lung biopsy to primary SBRT initiation were summarized among patients who received lung biopsy before SBRT initiation. All analyses were conducted in SAS version 9.4 (SAS Institute Inc., Cary, NC, USA) and R version 3.4.2 (R Foundation for Statistical Computing, Vienna, Austria).

## 3. Results

### 3.1. Patient Characteristics

The study included 3014 patients who had stage I-IIB (N0) NSCLC and met all criteria; the median follow-up time was 2.9 years (Figure 1). Among these patients, 2302 (76.4%), 454 (15.1%), 168 (5.6%), and 90 (3.0%) were diagnosed with stage IA, IB, IIA, and IIB disease, respectively, at initial NSCLC diagnosis. The mean age at diagnosis was 77.3 years, 37.7% of patients were male, and 86.9% were White. Slightly over half (50.6%) of the patients had non-squamous disease at initial diagnosis and the mean CCI score was 2.4 (Table 1).

### 3.2. Survival Outcomes

#### 3.2.1. Real-World Event-Free Survival

**Figure 2:**
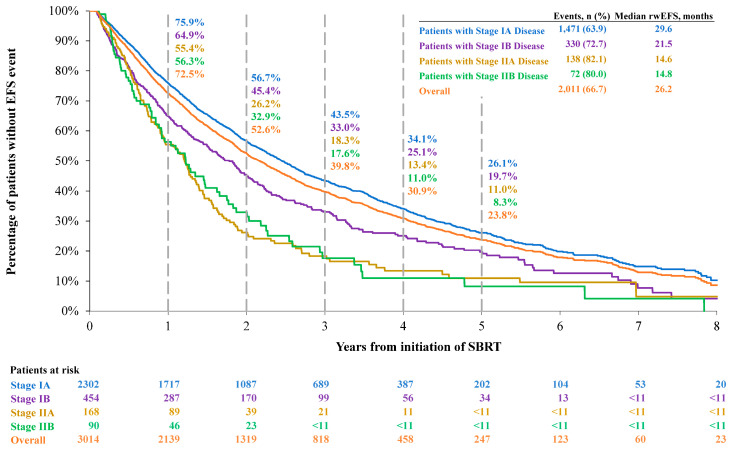
Real-world event-free survival during the study period. Abbreviations: rwEFS, real-world event-free survival; SBRT, stereotactic body radiotherapy. Note: numbers < 11 and numbers that can be used to derive N < 11 are not reported to comply with the data suppression policy from SEER-Medicare.

The median rwEFS was 26.2 months and the 1-, 3-, and 5-year rwEFS rates were 72.5%, 39.8%, and 23.8%, respectively, for the overall population (Figure 2). Patients who were initially diagnosed with more advanced NSCLC had generally shorter rwEFS. Specifically, for patients with stage IA, IB, IIA, and IIB disease, the median rwEFS was 29.6, 21.5, 14.6, and 14.8 months, and the 5-year rwEFS rates post-SBRT were 26.1%, 19.7%, 11.0%, and 8.3%, respectively.

#### 3.2.2. Overall Survival

**Figure 3:**
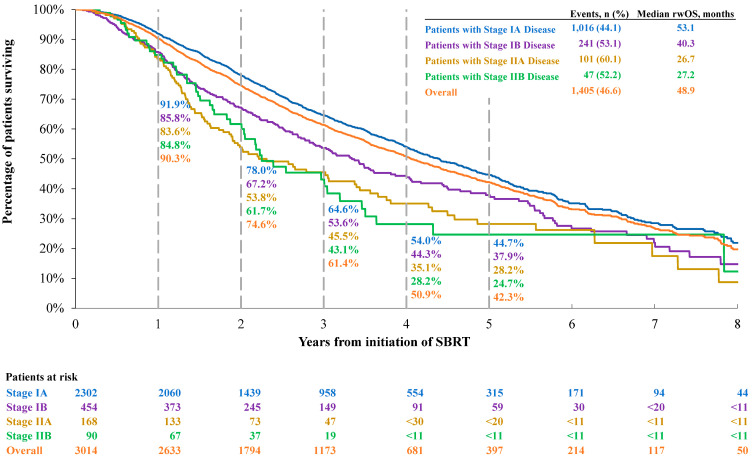
Real-world overall survival by disease stage. Abbreviations: OS, overall survival; SBRT, stereotactic body radiotherapy. Note: numbers < 11 and numbers that can be used to derive N < 11 are not reported to comply with the data suppression policy from SEER-Medicare.

In the overall population, the median OS was 48.9 months while the 1-, 3-, and 5-year OS rates were 90.3%, 61.4%, and 42.3% (Figure 3). Similarly to the findings for rwEFS, patients initially diagnosed with more advanced NSCLC had generally shorter OS. The median OS was 53.1, 40.3, 26.7, and 27.2 months for patients with stage IA, IB, IIA, and IIB disease, respectively, while the 5-year OS rates post-SBRT were 44.7%, 37.9%, 28.2%, and 24.7%.

#### 3.2.3. Cumulative Incidence of Lung Cancer-Specific Death

**Figure 4:**
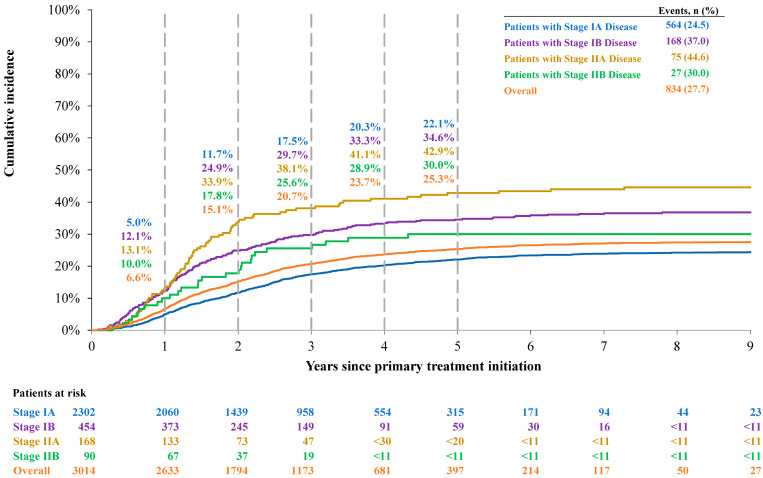
Cumulative incidence of lung cancer-specific death by disease stage. Note: numbers < 11 and numbers that can be used to derive N < 11 are not reported to comply with the data suppression policy from SEER-Medicare.

The cumulative incidence of lung cancer-specific death for the overall population was 6.6% at 1 year, 20.7% at 3 years, and 25.3% at 5 years (Figure 4). For patients with stage IA, IB, IIA, and IIB disease at initial diagnosis, the 5-year cumulative incidence rates of lung cancer-specific death were 22.1%, 34.6%, 42.9%, and 30.0%, respectively.

#### 3.2.4. TDDM

In the overall population, the median TDDM was 30.9 months, and the 1-, 3-, and 5-year TDDM rates were 77.1%, 44.7%, and 27.4%, respectively (Supplementary Figure S1). For patients with stage IA, IB, IIA, and IIB disease, the median TDDM was 34.5, 24.9, 15.3, and 19.6 months, respectively, and the 5-year TDDM rates post-SBRT were 30.0%, 21.8%, 12.9%, and 13.3%, respectively.

### 3.3. Lung Biopsy Patterns

Of the overall patient population (N = 3014), 2715 (90.1%) underwent a biopsy within 12 months before primary SBRT initiation (Table 2). The remaining patients (N = 299, 9.9%) did not undergo biopsy during the 12 months before primary SBRT initiation. For patients who underwent lung biopsy within 12 months before primary SBRT initiation, percutaneous biopsy was the most common procedure (2252 [82.9%]), followed by bronchial biopsy (794 [29.2%]). Additionally, 539 (19.9%) patients underwent fine needle aspiration. In this group, the mean and median times from first lung biopsy to SBRT initiation were 68.8 and 49.0 days, respectively, with 1982 (75%) patients undergoing the procedure within 73 days before primary SBRT initiation.

## 4. Discussion

This US retrospective observational study described the real-world survival outcomes among older patients with unresected NSCLC who received SBRT, as well as the utilization of lung biopsy prior to and after SBRT. The rwEFS and OS rates after SBRT generally declined among patients diagnosed with more advanced NSCLC. Among patients with a death event, more than half died from non-lung cancer causes. The median rwEFS and OS for the overall patient population were 2.2 and 4.1 years, respectively, but were nearly halved (1.2 and 2.3 years) for patients with stage IIB disease at diagnosis. Given the lack of recent real-world data on clinical outcomes by disease stage among US patients with unresected NSCLC receiving SBRT, these results benchmark the outcomes of this difficult-to-treat population.

The present findings are largely aligned with those of prospective NSCLC clinical trials assessing survival outcomes in patients with unresected disease who received SBRT. In the phase II NRG Oncology RTOG 0236 trial of SBRT in patients with medically inoperable NSCLC, the median disease-free survival (DFS) and OS were 3.0 years and 4.0 years, and DFS and OS at 5 years were 25.5% and 40.0%, respectively [9]. In comparison, 5-year rwEFS and OS were 23.8% and 42.3%, respectively, in the present study’s overall population. However, the OS of the overall population of this study is somewhat higher than that reported in the two previous real-world studies of patients with NSCLC undergoing SBRT, likely due to differences in patient populations and the use of more recent data in this study. For example, a single-center registry study by Arnett et al. reported that the median and 5-year OS in stage IA to IIB NSCLC treated by SBRT (2008–2014) were 3.5 years and 35%, respectively [18]. This is lower than the present results, although the differences could be attributed to our focus on patients with newly diagnosed NSCLC while Arnett et al. included patients with progressed NSCLC. Based on more recent data from a single institution, Ji et al. reported a median PFS of 31 months and a median OS of 69 months in patients aged ≥65 years diagnosed with stage I-II NSCLC between 2009 and 2020 [11]. Additionally, a study by Chi et al. using data from the US National Cancer Database reported that the 5-year OS for patients with stage IA-III NSCLC diagnosed between 2004 and 2015 and receiving SBRT was 30.4% [19]. Approximately 40% of patients in Chi et al. had stage I disease at initial diagnosis, compared with >76% in the current study, likely explaining their comparatively lower 5-year OS. Furthermore, compared to the present study, both Arnett et al. and Chi et al. differed in the timeframe of assessment (i.e., 2007–2020 in the present study) and there may have been evolution in clinical practice in more recent years.

The current findings on the cumulative incidence of lung cancer-specific death among older patients with unresected NSCLC who received primary SBRT in the US (i.e., 5-year cumulative incidence of 25.3%) are largely aligned with the results of prior studies assessing lung cancer-specific survival. For example, the reported 3-year lung cancer-specific survival rates have ranged from 77−94% for patients with stage I NSCLC receiving SBRT [7,13,20], indicating that 6–23% of patients died because of lung cancer by year 3. A study by Tran et al. using SEER-Medicare data (2005–2015) reported that the 1-, 3-, and 5-year lung cancer-specific survival was 76%, 51%, and 46%, respectively, in patients with stage IIA (per AJCC 7th edition) N0 NSCLC tumors >5 cm who received SBRT, and a median lung cancer-specific survival of 26.3 months [21], indicating that 24%, 49%, and 54% of patients had a lung cancer-specific death event by year 1, 3, and 5 after treatment initiation. In comparison, the 5-year cumulative incidence of lung cancer-specific death for stage IIA (per AJCC 8th edition) patients diagnosed between 2007 and 2019 in this study was 42.9%. As prior studies have primarily focused on patients with specific disease stages, this study contributes to the existing literature by assessing the cumulative incidence of lung cancer-specific death among a broader population with unresected NSCLC who received primary SBRT with more recent data in the real world. This study also adds novel findings on TDDM, an outcome that has not been previously explored in this population. The overall 5-year TDDM rate was 27.4%, with variation across disease stages.

Efforts to augment regional and distant disease control following SBRT have focused on integrating immunotherapy, chemotherapy, and targeted therapies. While many medically inoperable patients are often unable to tolerate chemotherapy, current National Comprehensive Cancer Network Clinical Practice Guidelines in Oncology (NCCN Guidelines^®^) recommended considering adjuvant chemotherapy following SBRT for high-risk stage II NSCLC [22]. Multiple randomized phase 3 trials have evaluated the integration of immunotherapy with SBRT for early-stage inoperable NSCLC (NCT04214262, NCT03833154, NCT03924869) [23,24,25]. However, two of these three trials were terminated early after interim analyses. As such, novel systemic strategies to augment SBRT in the early-stage, inoperable population are warranted.

In the assessment of lung biopsy utilization, the results indicated that the large majority (>90%) of patients received lung biopsy before primary SBRT initiation, which was, on average, 68.8 days before SBRT initiation. This is an encouraging finding and reflects the recommendations of current treatment guidelines in NSCLC from the American Society of Clinical Oncology and American Society for Radiation Oncology to obtain a tissue diagnosis when feasible, reserving empiric SBRT for cases in which percutaneous or endobronchial biopsy is deemed unsafe [15]. There is very limited real-world evidence with which to compare these findings, although a study using data from the US National Cancer Database (2003–2011) similarly reported that 95.5% of patients with stage I NSCLC who received SBRT had biopsy before SBRT, and patients who did not receive biopsy were diagnosed based on radiographic evidence or clinical suspicion [26].

The results of this study should be considered in light of several limitations, some of which are inherent to claims database studies (e.g., misclassification bias stemming from coding errors or data omissions, which may affect data accuracy). First, the linked SEER-Medicare database contains information on Medicare patients who are all 65 years or older. Therefore, the study findings may not be representative of the clinical outcomes or biopsy use among younger patients. Second, due to the nature of administrative claims data, clinical rationale behind treatment decisions could not be captured; future research with more granular clinical data may help us better understand the treatment decisions for patients with unresected stage I-IIB (N0) NSCLC, such as reasons for not pursuing surgery. Further, disease recurrence status cannot be identified directly from claims data; as such, this study used an algorithm that incorporated combinations of procedure, diagnosis, and drug codes, along with predefined assumptions. This approach, while previously used in the literature, may have limited sensitivity and requires further validation. For example, coding inaccuracies could have introduced misclassification and misidentification of patients with NSCLC recurrence. Similarly, biopsy use was determined from Medicare claims data, which may be subject to these same errors. Third, the study focused on patients with confirmed diagnosis of NSCLC before SBRT initiation, which may lead to an overestimation of biopsy rates. Lastly, while the study used US Food and Drug Administration-approved and guideline-recommended treatments for advanced NSCLC as an indicator for disease recurrence, it is possible that these treatments were administered for other primary cancers that developed after NSCLC.

## 5. Conclusions

Among older US patients with unresected, stage I-IIB (N0) NSCLC treated with primary SBRT, more than 50% experienced disease recurrence or death within 3 years after SBRT initiation. The median OS was 48.9 months post-treatment and the 5-year OS rate was 42.3%; approximately 25% of patients died within 5 years after SBRT initiation due to lung cancer, suggesting a high rate of death from competing risks. Further, patients who had more advanced NSCLC stage at initial diagnosis had inferior survival outcomes. Together, these results benchmark the real-world outcomes in this difficult-to-treat population for future studies assessing novel treatment strategies that can further augment outcomes in this patient population.

## Figures and Tables

**Table 1:** Baseline characteristics of patients with NSCLC, overall and by disease stage.

	Patients with Stage IA Disease (N = 2302)	Patients with Stage IB Disease (N = 454)	Patients with Stage IIA Disease (N = 168)	Patients with Stage IIB Disease (N = 90)	Overall Patients (N = 3014)
**Demographic characteristics**					
Age at diagnosis (years), mean ± SD	77.0 ± 6.4	78.1 ± 6.7	78.4 ± 7.1	79.3 ± 6.7	77.3 ± 6.6
Male, N (%)	835 (36.3%)	189 (41.6%)	65 (38.7%)	48 (53.3%)	1137 (37.7%)
Race/ethnicity, N (%)					
White	2006 (87.1%)	396 (87.2%)	139 (82.7%)	79 (87.8%)	2620 (86.9%)
Black	132 (5.7%)	>18 ^a^	13 (7.7%)	<11 ^a^	173 (5.7%)
Hispanic	66 (2.9%)	14 (3.1%)	<11 ^a^	<11 ^a^	87 (2.9%)
Asian	82 (3.6%)	15 (3.3%)	<11 ^a^	<11 ^a^	111 (3.7%)
Other	16 (0.7%)	<11 ^a^	<11 ^a^	<11 ^a^	23 (0.8%)
**Clinical characteristics**					
Histology type, N (%)					
Squamous	727 (31.6%)	174 (38.3%)	79 (47.0%)	42 (46.7%)	1022 (33.9%)
Non-squamous	1182 (51.3%)	225 (49.6%)	>70 ^a^	>35 ^a^	1525 (50.6%)
NOS	393 (17.1%)	55 (12.1%)	>15 ^a^	<11 ^a^	467 (15.5%)
SBRT index year, N (%)					
2007–2011	328 (14.2%)	59 (13.0%)	26 (15.5%)	14 (15.6%)	427 (14.2%)
2012–2016	1008 (43.8%)	220 (48.5%)	93 (55.4%)	33 (36.7%)	1354 (44.9%)
2017–2020	966 (42.0%)	175 (38.5%)	49 (29.2%)	43 (47.8%)	1233 (40.9%)
CCI, mean ± SD	2.4 ± 1.7	2.5 ± 1.8	2.7 ± 1.9	2.4 ± 1.7	2.4 ± 1.7

Abbreviations: CCI, Charlson Comorbidity Index; NOS, not otherwise specified; NSCLC, non-small-cell lung cancer; SBRT, stereotactic body radiotherapy; SD, standard deviation. Note: ^a^ numbers < 11 and numbers that can be used to derive N < 11 are not reported to comply with the data suppression policy from SEER-Medicare.

**Table 2:** Distribution of patients who received lung biopsy by biopsy type ^a^.

Biopsy Type	Patients Who Received Biopsy Within 12 Months Before Primary SBRT Initiation
Total	2715 (90.1%)
Lung biopsy, N (%)	
Fine needle biopsy	539 (19.9%)
Percutaneous biopsy	2252 (82.9%)
Surgical biopsy	<11 ^b^
Bronchial biopsy	794 (29.2%)
Other lung biopsy	>68 ^b^

Abbreviation: SBRT, stereotactic body radiotherapy. Notes: ^a^ patients might receive one or multiple biopsy procedures, so the numbers and percentages across rows do not add up. A total of 1614 patients received only one biopsy procedure type; the remaining 1101 patients received more than one biopsy procedure type. ^b^ Characteristics with N < 11 are not reported to comply with data suppression policy from SEER-Medicare.

## Data Availability

Data used in this study were obtained from SEER-Medicare. Applications are reviewed and approvals granted subject to conditions set forth by SEER-Medicare. Information on access procedures, general enquiries, and contact information can be found online. This study used the linked SEER-Medicare database. The interpretation and reporting of these data are the sole responsibility of the authors. The authors acknowledge the efforts of the National Cancer Institute; Information Management Services (IMS), Inc.; and the Surveillance, Epidemiology, and End Results (SEER) Program tumor registries in the creation of the SEER-Medicare database. In addition, the collection of cancer incidence data used in this study was supported by the California Department of Public Health pursuant to California Health and Safety Code Section 103885; Centers for Disease Control and Prevention’s (CDC) National Program of Cancer Registries, under cooperative agreement 1NU58DP007156; the National Cancer Institute’s Surveillance, Epidemiology and End Results Program under contract HHSN261201800032I awarded to the University of California, San Francisco, contract HHSN261201800015I awarded to the University of Southern California, and contract HHSN261201800009I awarded to the Public Health Institute. The ideas and opinions expressed herein are those of the author(s) and do not necessarily reflect the opinions of the State of California, Department of Public Health, the National Cancer Institute, and the Centers for Disease Control and Prevention or their contractors and subcontractors.

## Supplementary Materials

The following supporting information can be downloaded at https://www.mdpi.com/article/10.3390/jcm14238604/s1, Table S1. List of codes considered in the study; Figure S1. Real-world time to death or distant metastasis by disease stage.
