# Supplementary material for: Real-World Clinical Outcomes and Biopsy Patterns of Older Patients with Unresected Non-Small-Cell Lung Cancer Treated with Primary Stereotactic Body Radiotherapy
Source: J Clin Med. 2025 Dec 4;14(23):8604. doi: 10.3390/jcm14238604 (PMC12692972; doi:10.3390/jcm14238604)
Supplement: Supplementary file 1 [file jcm-14-08604-s001.zip › jcm-3988354-supplementary.pdf]

Supplementary Material *for* Real-World Clinical Outcomes and Biopsy Patterns of Older Patients with Unresected Non-Small-Cell Lung Cancer Treated with Primary Stereotactic Body Radiotherapy

Supplementary Table S1. List of Codes Considered in the Study

**Diagnosis or procedure**	**Codes**
**NSCLC**	ICD-O3 codes 340-C343, C348, and C349, with the relevant histology codes 8010, 8012, 8013, 8020, 8046, 8050–8052, 8070–8078, 8140, 8141, 8143, 8147, 8250–8255, 8260, 8310, 8430, 8480, 8481, 8490, 8560, 8570–8575
**SBRT**	ICD-9-PCS: 923.x<br>ICD-10-PCS: DB2*<br>CPT codes: 32701, 77373, 77435, G0173, G0251, G0339, G0340

Abbreviations: CPT, Current Procedural Terminology; ICD-9/10-PCS International Classification of Diseases, 9th/10th Revision, Procedure Coding System ICD-O3, International Classification of Diseases for Oncology, 3rd edition; NSCLC, non-small cell lung cancer; SBRT, stereotactic body radiation therapy.

Supplementary Figure S1. Real-World Time to Death or Distant Metastasis by Disease Stage[1,2,3]

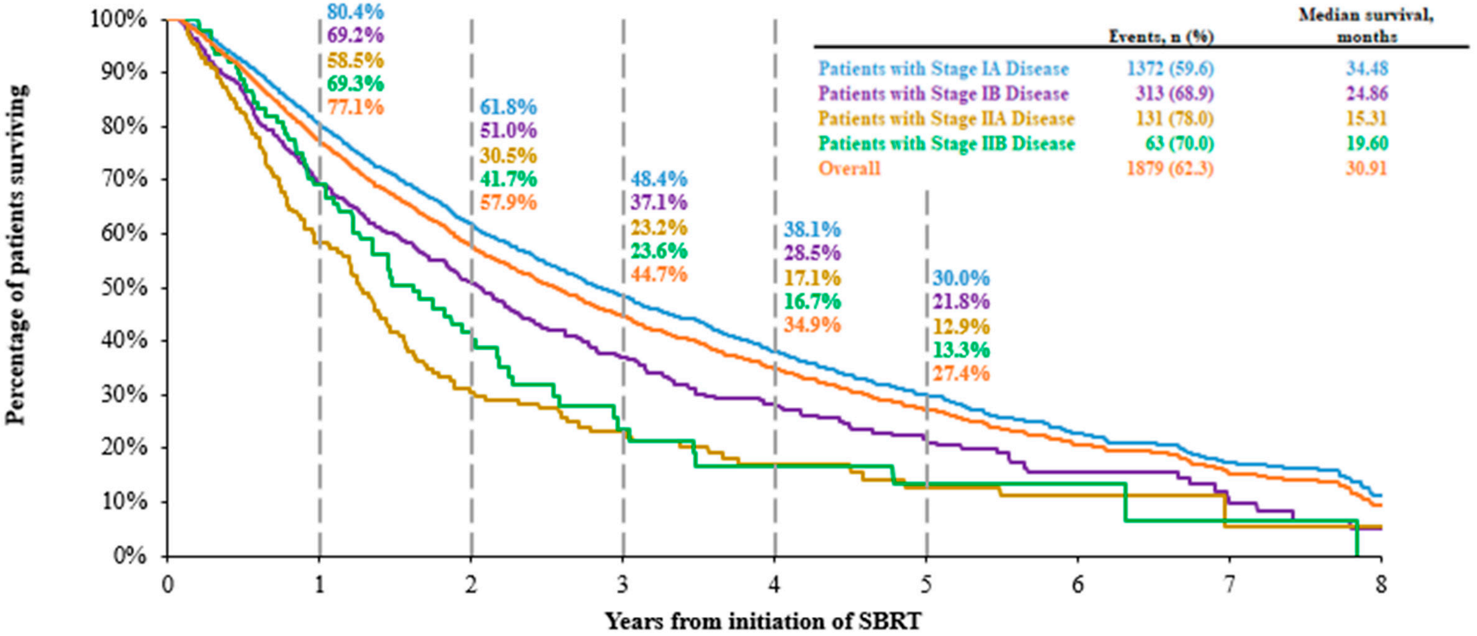

Patients at risk

Stage IA	2302	1814	1175	758	422	225	119	64	22
Stage IB	454	306	189	109	60	36	16	<11	<11
Stage IIA	168	93	44	25	<15	<11	<11	<11	<11
Stage IIB	90	56	27	11	<11	<11	<11	<11	<11
Overall	3014	2269	1435	903	500	273	142	73	26

Abbreviation: SBRT, stereotactic body radiation therapy. **Notes:** [1] Numbers <11 and numbers that can be used to derive N<11 are not reported to comply with the data suppression policy from SEER-Medicare. [2] Patients were followed from the initiation of SBRT until the earliest of 1) death, 2) end of Medicare Part A, B, or D eligibility, or 3) end of data availability on December 31, 2020. [3] Survival time was calculated as the time from the initiation of SBRT to the date of first distant metastasis or death. Patients who did not have a recorded death date after the initiation of neoadjuvant therapy date were censored at the last day of follow-up.
